# Interferon-related inflammaging links epigenetic age acceleration to multimorbidity

**DOI:** 10.1016/j.xgen.2026.101218

**Published:** 2026-04-17

**Authors:** Zhaoli Liu, Athanasios Ziogas, Yihan Zhang, Manoj Kumar Gupta, Konstantin Föhse, Esther Taks, Elisabeth Dulfer, Andrei Sarlea, Lorenzo Ventriglia, Büsra Geckin, Mohamad Ballan, Nienke van Unen, Leonie Helder, Stephanie Trittel, Peggy Riese, Simone Moorlag, Charlotte de Bree, Valerie Koeken, Vera Mourits, Martin Jaeger, Frank Pessler, Carlos A. Guzmán, Leo A.B. Joosten, Yang Li, Cheng-Jian Xu, Mihai G. Netea

**Affiliations:** 1 Centre for Individualised Infection Medicine (CiiM), a Joint Venture Between the Helmholtz Centre for Infection Research (HZI) and Hannover Medical School (MHH), Hannover, Germany; 2 TWINCORE, a Joint Venture Between the Helmholtz Centre for Infection Research (HZI) and Hannover Medical School (MHH), Hannover, Germany; 3 Department of Internal Medicine and Radboudumc Community for Infectious Diseases (RCI), Radboud University Medical Center, Nijmegen, the Netherlands; 4 Department of Precision Medicine, University of Campania “Luigi Vanvitelli,” Naples, Italy; 5 Department of Vaccinology and Applied Microbiology, Helmholtz Centre for Infection Research, Braunschweig, Germany; 6 Research Group Biomarkers for Infectious Diseases, TWINCORE Centre for Experimental and Clinical Infection Research, Hannover, Germany; 7 Department of Medical Genetics, Iuliu Hațieganu University of Medicine and Pharmacy, Cluj-Napoca, Romania; 8 Cluster of Excellence RESIST (EXC 2155), Hanover Medical School, Hannover, Germany; 9 Lower Saxony Center for Artificial Intelligence and Causal Methods in Medicine (CAIMed), Hannover, Germany; 10 Department for Immunology and Metabolism, Life and Medical Sciences Institute (LIMES), University of Bonn, Bonn, Germany; 11 Human Genomics Laboratory, University of Medicine and Pharmacy of Craiova, Craiova, Romania; 12 National Health Commission Key Laboratory of Cardiovascular Regenerative Medicine, Central China Subcenter of National Center for Cardiovascular Diseases, Henan Cardiovascular Disease Center, Fuwai Central-China Cardiovascular Hospital, Central China Fuwai Hospital of Zhengzhou University, Zhengzhou, China; 13 Biomedical Research Institute, Foundation for Research and Technology, Ioannina, Greece; 14 Laboratory of Biological Chemistry, Department of Medicine, School of Health Sciences, University of Ioannina, Ioannina, Greece

**Keywords:** biological aging, inflammaging, epigenetic age acceleration, immunosenescence, frailty, multimorbidity

## Abstract

Chronic systemic inflammation and DNA methylation changes are two major hallmarks of aging, yet their interaction is poorly known. We investigated the relation between circulating inflammatory proteome and epigenetic age acceleration as assessed by DNA methylation in four independent cohorts of different ages and health conditions. Epigenetic age scores known to predict human health span (GrimAge and PhenoAge) were more strongly associated with age-associated inflammatory proteins, frailty, and multimorbidity when compared to epigenetic age scores associated with lifespan (Horvath and Hannum). Mendelian randomization analyses showed that blood concentrations of important inflammatory cytokines associated with the interferon pathway (CXCL9, CXCL10, CCL11, and IL-18) increase with age and are causal drivers of epigenetic age acceleration and age-related diseases. Furthermore, aging was associated with dysregulation of cytokine production capacity in immune cells in response to microbial stimulation. These findings argue that the interferon pathway may represent a target for anti-aging interventions.

## Highlights

• Health span clocks link more strongly to inflammation than lifespan clocks
• Interferon-related inflammatory proteins causally drive epigenetic age acceleration
• The interferon pathway is a potential target for anti-aging therapies

## Graphical abstract

**Figure:**
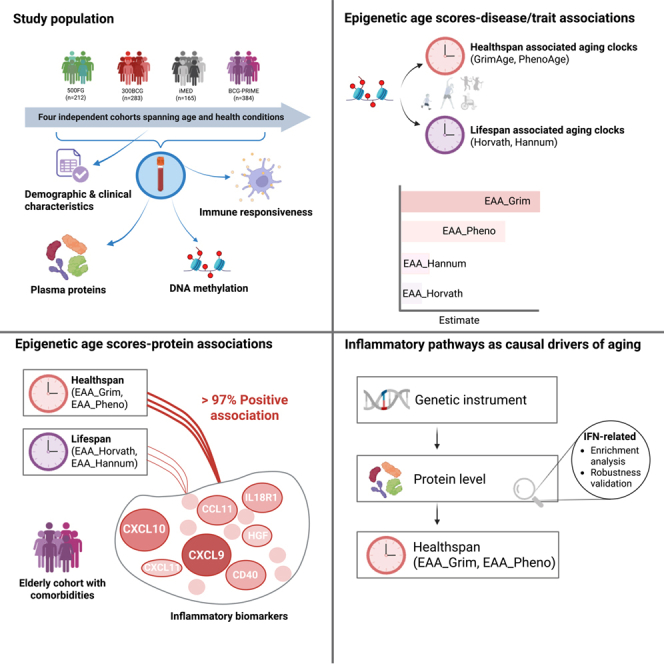

## Teaser

Liu et al. investigated the relationship between two hallmarks of aging (chronic inflammation and DNA methylation alterations) in four independent cohorts covering different ages and health conditions. They found that inflammatory proteins, which increase with age and are related to the interferon signaling pathway, causally drive epigenetic age acceleration.

## Introduction

Aging is a major risk factor for numerous diseases, including infections, diabetes, gout, neurodegeneration, cardiovascular diseases, and cancer.^1^^,^^2^ Therefore, it is crucial to understand the pathophysiological processes leading to biological aging in order to design novel therapies to improve health in the elderly population. Studies performed during the last two decades have identified important biological processes that change during old age and are likely involved in the pathophysiology of aging, with 12 major hallmarks of aging being proposed: genomic instability, telomere attrition, epigenetic alterations, loss of proteostasis, disabled macroautophagy, deregulated nutrient sensing, mitochondrial dysfunction, cellular senescence, stem cell exhaustion, altered intercellular communication, chronic inflammation, and dysbiosis.^3^ However, not every individual ages in a similar way, and susceptibility to age-related diseases varies widely in the population. While chronological age is a strong predictor of disease risk, increasing evidence argues that the biological age of the organism, assessed through molecular and physiological biomarkers, may provide a significantly better reflection of the aging process and the risk of age-associated diseases.^4^^,^^5^

One of the most robust aging-associated molecular changes is the alteration of DNA methylation patterns in cells of individuals of old age.^3^^,^^6^ Epigenetic clocks, which assess DNA methylation status at specific sites, have been developed to both reflect chronological age and provide an estimate of the biological age to predict mortality risk.^5^ Notable examples of these clocks include Horvath’s clock^7^ and Hannum’s clock,^8^ which have shown very good correlations with the lifespan of individuals. On the other hand, other epigenetic age (EpiAge) clocks, such as PhenoAge^9^ and GrimAge,^10^ are effective biological age estimators for predicting health span and susceptibility to diseases.^10^^,^^11^ The difference between DNA methylation age and chronological age is referred to as epigenetic age acceleration (EAA). EAA is typically calculated as the residuals from regressing DNA methylation age on chronological age. A positive EAA indicates that an individual’s biological age is older than their chronological age, while a negative EAA suggests their aging process is slower than expected.^11^ However, little is known regarding the biological pathways leading to EAA in some individuals of older age, which therefore limits the capacity to modulate it.

Immune mechanisms play a crucial role in most age-associated diseases, and two main alterations are associated with an aged immune system: a process of low-grade chronic systemic inflammation (also called “inflammaging”) and a defective responsiveness of the immune system to stimulation (termed “immunosenescence”). Chronic low-grade inflammation increases with age and contributes to the development of age-related diseases.^12^ A previous study suggested that reducing inflammation or targeting its causes, such as through antiviral treatment, may slow the biological aging assessed by DNA methylation.^13^ However, the potential causal role of inflammation in biological aging, particularly in driving EAA, remains largely unknown.

In this study, we assessed the relationship between biological aging as mirrored by epigenetic aging scores and the defects of immune responses associated with aging: inflammaging and immunosenescence. We analyzed data from four cohorts to explore the relationships between a comprehensive set of circulating inflammatory biomarkers and chronological age, EpiAge scores, and EAA. Using Mendelian randomization (MR), we also investigated the potential causal links between inflammatory proteins/pathways and EAA and the impact of these processes on immune responsiveness to microbes, general frailty, and the presence of chronic non-communicable diseases. Our study helps understand the causal role of inflammation as a crucial factor driving EAA and is important for identifying potential targets for anti-aging interventions.

## Results

### EpiAge scores are associated with age-related diseases

**Figure 1:**
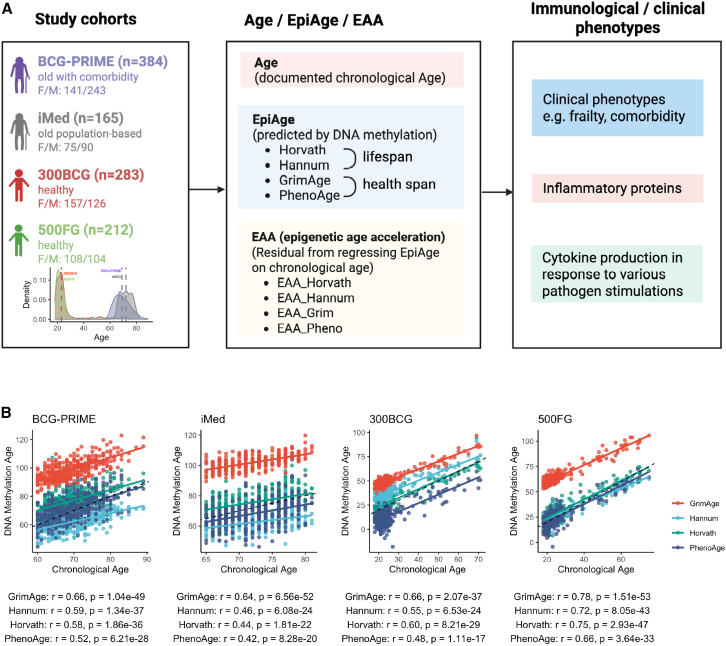
Study design and epigenetic age (A) Study design. The figure was generated by BioRender.com. (B) Scatterplot illustrating the correlation between DNA methylation age and chronological age. Spearman correlation analysis was performed to assess the associations. The sample size for each cohort is as follows: 300BCG, *n* = 283; 500FG, *n* = 212; iMED, *n* = 165; and BCG-PRIME, *n* = 384.

Using DNA methylation profiles derived from whole blood, we assessed several epigenetic aging scores in four cohorts of individuals with diverse age and health conditions: BCG-PRIME (*n* = 384), iMed (*n* = 165), 300BCG (*n* = 283), and 500FG (*n* = 212) cohorts (Figure 1A). The BCG-PRIME and iMed cohorts comprised individuals aged 60 years and above (mean ages: BCG-PRIME, 69 years and iMed, 72 years). 300BCG and 500FG were mainly composed of young, healthy individuals with a mean age of 25 years. DNA methylation profiles from these cohorts were analyzed to estimate EpiAge using lifespan-associated Horvath and Hannum methylation age clocks, as well as health-span-associated GrimAge and PhenoAge methylation age clocks. Overall, DNA methylation profiles at CpG sites included in the epigenetic aging clock models showed no systematic directional shift across cohorts of different ages and conditions. However, significant CpGs were enriched in CpG islands, N_Shelf, S_Shelf, and OpenSea regions but depleted in N_Shore and S_Shore regions (Figures S1A and S1B). In all four cohorts, EpiAge showed a significant correlation with documented age (Figure 1B). The discrepancy between EpiAge and chronological age was calculated as EAA.

**Figure 2:**
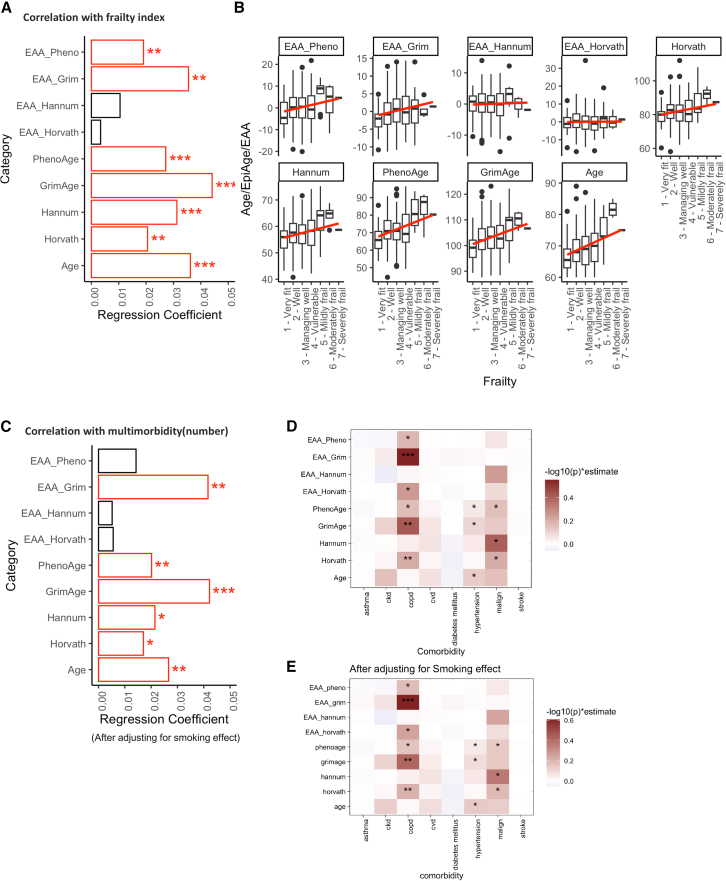
Aging links to the frailty index and multimorbidity (A) Barplot showing the correlation between age-related measures and frailty index. Statistically significant correlations after multiple testing are denoted as follows: ∗*p*adj < 0.05, ∗∗*p*adj < 0.01, and ∗∗∗*p*adj < 0.001. (B) Boxplot detailing the correlation between each age-related measure and frailty index. (C) Barplot showing the correlation between age-related measures and the number of morbidities. Statistically significant correlations after multiple testing are denoted as follows: ∗*p*adj < 0.05, ∗∗*p*adj < 0.01, and ∗∗∗*p*adj < 0.001. (D) Correlation heatmap showing the associations between age-related phenotypes and the presence of morbidity. Each cell represents the correlation coefficient, with the color intensity reflecting the strength and direction of the correlation, calculated as −log10(nominal *p*) × sign(estimate). Statistically significant correlations after multiple testing are denoted as follows: ∗*p*adj < 0.05, ∗∗*p*adj < 0.01, and ∗∗∗*p*adj < 0.001. (E) Correlation heatmap showing the associations between age-related phenotypes and the presence of morbidity after adjusting for smoking effect. A linear regression model was fitted to evaluate these associations, and the analyses were done on the BCG-PRIME cohort (*n* = 384).

To validate the relevance of the epigenetic aging scores for the health status and age-related pathologies of the individuals, we conducted an association analysis between EpiAge clocks with the frailty index and the presence of chronic non-communicable diseases using data from the BCG-PRIME cohort, in which all individuals had at least one chronic disease (comorbidity). First, we observed significant positive correlations between most age-related phenotypes (except EAA_Horvath and EAA_Hannum) and frailty (Figures 2A and 2B). Furthermore, we found that age/EpiAge/EAA_Grim were positively correlated with the total number of age-related diseases (multimorbidity), indicating a strong association with multimorbidity (Figure 2C). Multimorbidity reflects the cumulative physiological damage in an individual and has become the key focus in aging research.^14^^,^^15^ The analysis included different diseases of different physiological systems: cancer, cardiovascular disease, stroke, diabetes mellitus, chronic obstructive pulmonary disease (COPD), asthma, chronic kidney disease (CKD), dementia, and others. Notably, chronological age is only associated with hypertension, whereas biological age is a stronger predictor not only for hypertension but also for COPD and malignancies. Both EAA and EpiAge, but not chronological age, were significantly associated with COPD (Figure 2D). This significant association was also observed after adjusting for the smoking effect (Figure 2E). These data demonstrate that EpiAge scores have important predictive value for frailty and age-related diseases, validating their use in the assessment of biological aging. Importantly, the well-known health-span-related GrimAge and PhenoAge epigenetic aging scores show stronger associations with age-related pathologies and frailty than lifespan-related Hannum and Horvath scores.

### Inflammatory pathways as causal drivers of aging

**Figure 3:**
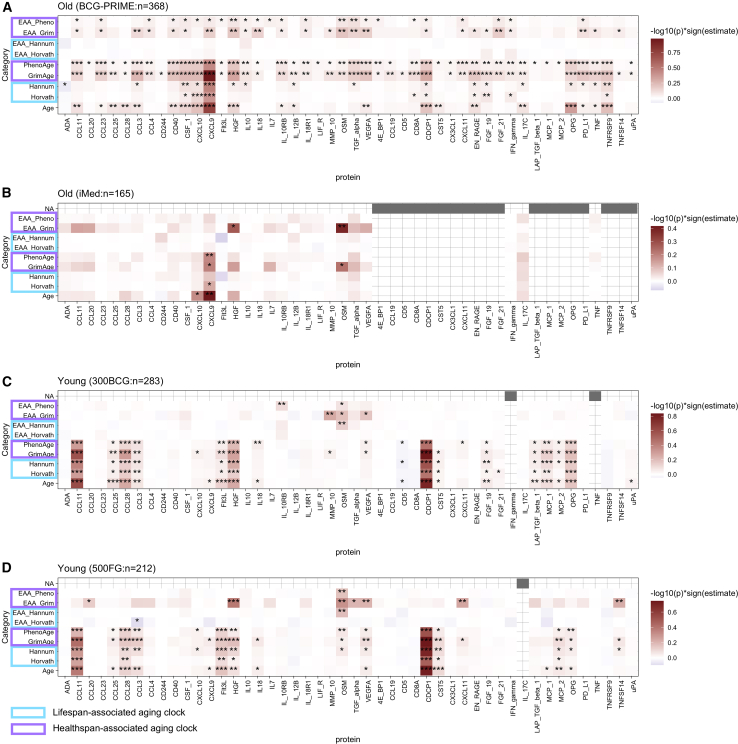
Inflammatory proteins are more strongly associated with health-span-related epigenetic clocks than with lifespan clocks in the old with comorbidities Correlation heatmap showing the associations between age-related phenotypes and inflammatory proteins across four cohorts. Association results in the (A) BCG-PRIME, (B) iMED, (C) 300BCG, and (D) 500FG cohorts. Each cell represents the correlation coefficient between a specific aging value and an inflammatory protein, with color intensity reflecting the strength and direction of the correlation, calculated as −log10(nominal *p*) × sign(estimate). Statistically significant correlations after multiple testing are denoted as follows: ∗*p*adj < 0.05, ∗∗*p*adj < 0.01, and ∗∗∗*p*adj < 0.001. A linear regression model was used to assess the statistical significance of the association. The sample size for each cohort is as follows: 300BCG, *n* = 283; 500FG, *n* = 212; iMED, *n* = 165; and BCG-PRIME, *n* = 384.

We subsequently analyzed the association between epigenetic aging scores, EAAs (EAA_Horvath, EAA_Hannum, EAA_Grim, and EAA_Pheno), and 64 (out of 92) circulating biomarkers of inflammation that passed the quality control criteria (at least 80% values measurable within the detection limit of the assay). First, we assessed this association in the BCG-PRIME cohort. Out of 576 tested associations, 231 reached nominal significance (*p* < 0.05). After adjusting for multiple testing, 179 associations achieved significant (adjusted p [*p*adj] < 0.05), involving 48 distinct proteins (Figure 3A; Table S1). Over 97% (47 of 48) of these inflammatory biomarkers showed a positive correlation with age-related phenotypes, especially with age, GrimAge, PhenoAge, EAA_Grim, and EAA_Pheno. The most significant association was observed between GrimAge and CXCL9 (*p*adj = 4.78 × 10^−13^). It is important to note that the EpiAge scores associated with health span, such as PhenoAge and GrimAge, exhibited stronger and more significant correlations with a broader range of inflammatory proteins than the lifespan-associated Horvath and Hannum clocks.

We extended the association analyses between aging scores and inflammatory proteins to three additional cohorts (Figures 3B–3D): one population-based elderly cohort (iMed) (Figure 3B) and two younger healthy cohorts (300BCG and 500FG) (Figures 3C and 3D; Tables S2, S3, and S4). Overall, most of the significant associations displayed a positive correlation pattern. Notably, CXCL9 and HGF showed significant correlations with chronological age across all four cohorts, consistent with findings from a previous study.^15^ Interestingly, our study further revealed that CXCL9 significantly correlated with EpiAge in the elderly cohorts (BCG-PRIME and iMed) and with EAA in the elderly cohort with comorbidities (BCG-PRIME) (Figures 3A and 3B).

**Figure 4:**
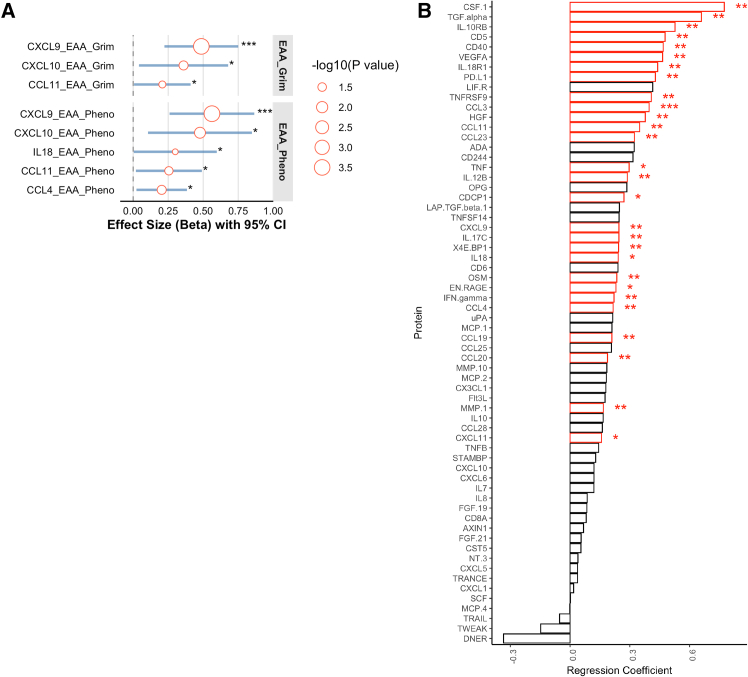
Key immunological biomarkers as causal drivers of aging (A) Forest plot illustrating the significant Mendelian randomization (MR) links between inflammatory protein and EAA using MR analysis, based on the inverse-variance-weighted method. (B) Barplot showing the correlation between inflammatory protein levels and the number of morbidities. Statistically significant correlations after multiple testing are denoted as follows: ∗*p*adj < 0.05, ∗∗*p*adj < 0.01, and ∗∗∗*p*adj < 0.001.

Next, we investigated whether inflammatory proteins might play a causal role in epigenetic acceleration (EAA_Pheno or EAA_Grim) using bidirectional two-sample MR.^16^ pQTL (protein quantitative trait locus) and EAA-GWAS (EAA genome-wide association study) results were derived from the publicly available summary statistics (pQTL: UKB_PPP,^17^ *n* = 33,529; EAA_GWAS,^18^ *n* = 34,710). There are no overlapping individuals between these two studies. We used GWAS summary statistics results from subjects of European ancestry to minimize potential confounding from population stratification. In total, 50 protein-EAA association pairs (25 proteins and two EAAs) were included in the MR analyses. In the forward MR analysis using clumped pQTLs (SNPs with *p* < 5 × 10^−8^ after clumping) as instrumental variables and EAA-GWAS as outcomes, we identified eight protein-EAA pairs showing nominal evidence of potential causal relationships (Figure 4A; Table S5). Specifically, CCL11, CXCL10, CXCL9, CCL4, and IL-18 were potentially causally associated with EAA_Pheno, while CCL11, CXCL9, and CXCL10 were potentially causally linked to EAA_Grim. Among these, the strongest causal relationship was observed between CXCL9 and EAA, suggesting a significant causal effect of CXCL9 on EAA, particularly EAA_Pheno (Figure 4A). Notably, this association remained statistically significant after correction for multiple testing using the Benjamini-Hochberg method (*p*adj_CXCL9-Pheno ivw_ = 0.006; *p*adj_CXCL9-Grim ivw_ = 0.007). Furthermore, the association between CXCL9 and EAA_Pheno demonstrated the strongest causal evidence across multiple MR methods: inverse-variance weighted (*p* = 2 × 10^−4^), weighted median (*p* = 7 × 10^−4^), weighted mode (*p* = 3 × 10^−3^), and Mendelian randomization pleiotropy residual sum and outlier (MR-PRESSO) (*p* = 2.1 × 10^−3^). Similarly, for EAA_Grim, CXCL9 showed consistent significant effects across weighted median (*p* = 0.03), weighted mode (*p* = 0.04), and MR-PRESSO (*p* = 2.3 × 10^−3^) methods. Sensitivity analyses further supported the robustness of this finding, showing no evidence of horizontal pleiotropy (MR-Egger intercept test *p* = 0.92) and minimal heterogeneity (Cochran’s Q *p* > 0.05). We further validated these results using *cis*-MR analysis using *cis*-pQTLs as instruments. CXCL9 consistently showed a causal association with EAA_Pheno (*p* = 0.002, inverse-variance-weighted method), while CXCL10, CXCL9, and TNF were causally linked to EAA_Grim. The consistent effects observed in both *trans*- and *cis*-MR analyses strengthen the evidence for a causal relationship. In the reverse MR analysis, when treating EAAs as exposures and inflammatory proteins as outcomes, only two associations reached marginal significance (EAA_Pheno on CCL23 and IL-7) (CCL23: *p* = 0.04; IL-7: *p* = 0.049), arguing against a major role for reverse causation.

The causal association between inflammatory biomarkers in the circulation and EAA suggested by MR argues that chronic systemic inflammation is a cause of age-related morbidities as well. Indeed, multimorbidity was positively correlated with circulating concentrations of inflammatory proteins (Figure 4B), indicating that inflammaging contributes to the age-related diseases.

To further identify upstream regulatory drivers of the proteins associated with EAA, we performed transcription factor enrichment analysis. Notably, IRF and STAT family transcription factors, including IRF1, STAT1, and STAT3, were significantly enriched regulators of the identified EAA-associated protein set (Figure S2A). These transcription factors are central mediators of interferon (IFN) signaling and key drivers of IFN-stimulated gene (ISG) expression. Then, we tested whether these significant proteins were enriched for ISGs. Among the significantly EAA-associated proteins, a disproportionate fraction belonged to ISGs, exceeding what would be expected by chance (Figures S2B and S2C).

### Sex-stratified association analysis between age-related phenotypes and inflammatory proteins

**Figure 5:**
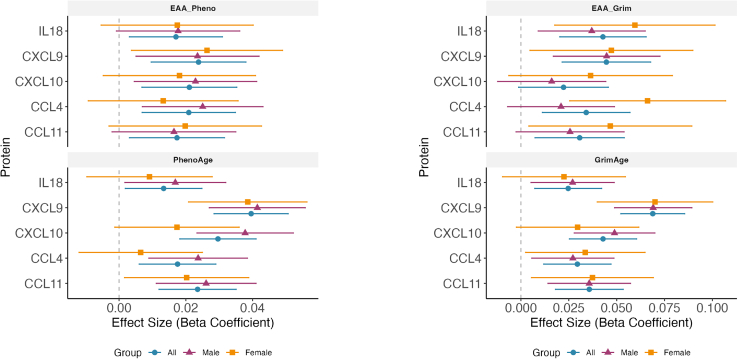
Sex-stratified analysis of the associations between inflammatory proteins and age-related phenotypes Forest plots showing the effect sizes (beta coefficients) and 95% confidence intervals from the linear regression models for the overall cohort, males, and females. The analysis included males (*n* = 243) and females (*n* = 141).

When stratified by sex, the direction of effects for EAA associations with CXCL9, CXCL10, CCL4, CCL11, and IL-18 remained aligned with those in the combined analysis. While statistical significance was attenuated in the smaller male and female subgroups, likely due to more limited sample sizes, the consistent effect directions support the robustness of our primary conclusions (Figure 5). The same pattern can also be observed for all proteins (Figures S3 and S4).

### Biological aging is associated with defective lymphocyte-derived cytokine production in response to pathogen stimulation

**Figure 6:**
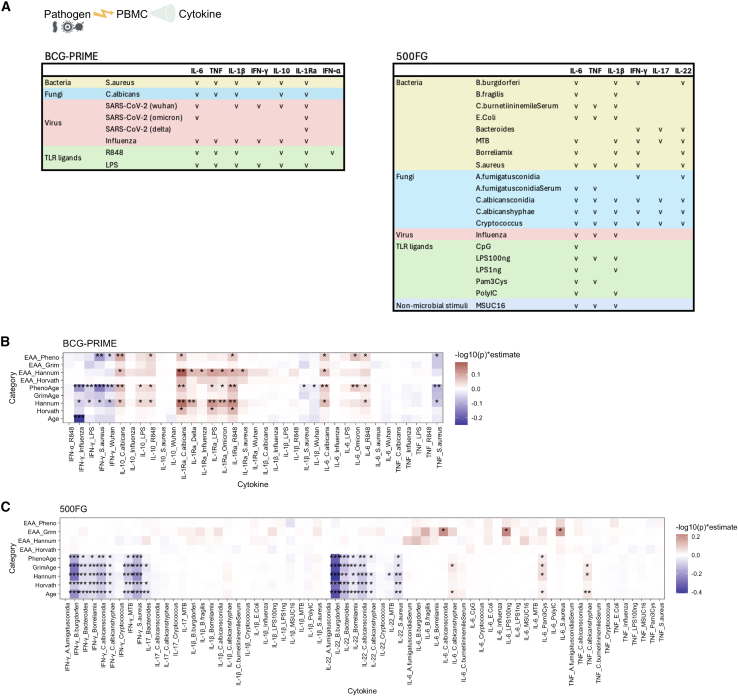
Aging links to cytokine production in response to pathogen stimulation (A) Plot illustrating stimulation experiments performed on human PBMCs using major human pathogens, followed by profiling of cytokines released into the serum. (B and C) Correlation heatmap showing associations between age-related phenotypes and cytokine production in response to pathogens in (B) BCG-PRIME and (C) 500FG cohorts. Each cell represents the correlation coefficient between a specific aging value and a cytokine production capacity, with color intensity reflecting the strength and direction of the correlation, calculated as −log10(nominal *p*) × sign(estimate). Statistically significant correlations after multiple testing are denoted as follows: ∗*p*adj < 0.05, ∗∗*p*adj < 0.01, and ∗∗∗*p*adj < 0.001. A linear regression model was used to assess the statistical significance of the association. The sample size for each cohort is as follows: 500FG, *n* = 212, and BCG-PRIME, *n* = 384.

In addition to its association with inflammaging and inflammatory diseases, aging is also accompanied by a decline in immune function and increased susceptibility to infections, a phenomenon known as immunosenescence. To investigate whether aging influences cytokine production in response to *ex vivo* stimulation with microbial stimuli, we also assessed cytokine production in peripheral blood mononuclear cells (PBMCs) from participants in the 500FG and BCG-PRIME cohorts. A broad range of pathogens, including bacterial, fungal, viral, and Toll-like receptor (TLR) ligands, were used for stimulation (Figure 6A). We observed that EpiAge scores were positively correlated with the production of mainly myeloid cell-derived cytokines such as IL-6 (in both BCG-PRIME and 500FG cohorts), as well as IL-10 and IL-1Ra (in the BCG-PRIME cohort) (Figures 6B and 6C). In contrast, negative associations were observed between epigenetic aging scores and lymphocyte-derived cytokines, including IFN-γ (in both cohorts) and IL-22 (in the 500FG cohort, where IL-22 was measured) upon various stimulations (Figures 6B and 6C). Specifically, consistent negative correlations were observed between IFN-γ production and both chronological age and EpiAge in the 500FG cohort, while these negative correlations were primarily observed with Hannum age, PhenoAge, and EAA_Pheno in the BCG-PRIME cohort. Our results showed that EAA may act as a more sensitive marker of biological aging and immune dysregulation in older individuals compared to young, healthy individuals.

## Discussion

Age is one of the most important risk factors for many diseases, yet not everyone ages in the same way. In recent years, the concept has been proposed that biological age is a better predictor of health and survival than simple chronological age,^19^^,^^20^ yet the best correlates for the biological age of an individual are still under debate. One of the most robust approaches to estimating biological age is measuring changes in DNA methylation during the process of aging.^21^ This approach is based on the observation that overall DNA methylation decreases during aging, contributing to the theory of “epigenetic loss of information” as a basis of biological aging.^22^ Several EpiAge scores have been subsequently developed, some of them predicting mainly the lifespan of an individual (Horvath score and Hannum score),^7^^,^^8^ while others also predict the health span (PhenoAge and GrimAge).^9^^,^^10^ The mechanisms that mediate the loss of DNA methylation during aging are poorly known, including a putative role of inflammation.^13^ Inflammation has been shown to contribute to accelerated aging, particularly in individuals with multimorbidity.^23^^,^^24^ In the present study, we aimed to investigate the potential relationship between chronic inflammation and EAA and to explore how age-related inflammation and DNA methylation changes connect to morbidity in elderly individuals.

As individuals age, external and internal factors lead to a process of chronic systemic inflammation that predisposes them to inflammatory, cardiovascular, and neurodegenerative diseases.^25^ At the same time, DNA methylation loss characterizes immune cells in the circulation of elderly individuals: whether epigenetic dysregulation leads to chronic inflammation or whether inflammation causes dysregulated DNA methylation patterns is not known. We have undertaken a systemic and comprehensive analysis of the association between inflammaging and EpiAge scores by investigating the circulating inflammatory proteome using a proximity extension assay, and the four main DNA methylation-based EpiAge scores associated with lifespan or health span (Horvath, Hannum, PhenoAge, and GrimAge) in four independent human cohorts.

First, we validated the well-known relationship between EAA and age-associated diseases in the BCG-PRIME cohort of elderly individuals, especially the scores previously reported to predict health span (PhenoAge and GrimAge).

Second, we observed a consistent positive correlation between plasma concentrations of inflammatory biomarkers and both chronological age and EpiAge in two cohorts of mostly young, healthy individuals (300BCG and 500FG). This positive association was also consistently observed in two cohorts of older individuals (more than 60 years of age), including one population based and one with comorbidities (BCG-PRIME cohort): this positive correlation was mainly observed between inflammatory proteins and epigenetic aging scores associated with health span (GrimAge, PhenoAge, Grim_EAA, and Pheno_EAA) rather than the epigenetic scores associated with lifespan (Horvath and Hannum). These findings suggest that in young, healthy cohorts, the relationship between inflammatory proteins and age-related measures is largely driven by normal aging processes. In contrast, in the older cohort with comorbidities, the stronger associations with GrimAge, PhenoAge, Grim_EAA, and Pheno_EAA may reflect the effects of inflammation on pathological aging. This aligns with the design of GrimAge and PhenoAge that integrate both chronological aging and pathological states,^26^^,^^27^ making them reflect not only chronological aging but also disease burden and mortality risk. Conversely, the weaker associations of Horvath_EAA and Hannum_EAA with markers of inflammation likely reflect their connection to intrinsic aging processes governed by genetic control.^5^

Third, we investigated the potential causality between chronic inflammation and EAA. Using MR, we identified key contributors with a putative causal role in driving EAA, including CXCL9, CXCL10, CCL11 (eotaxin), and IL-18. Previous studies have highlighted these proteins linked to age or age-related conditions. For example, CXCL9 serves as a strong predictor of an inflammatory aging clock and a marker of cardiovascular pathology.^15^ IL-18 was reported to be a mediator of inflammation involved in the aging process.^28^ CXCL10 concentrations have been shown to increase with age, and the DNA methylation level in the CXCL10 promoter region decreased with age.^29^ Elevated CCL11 circulating concentrations in old age have been linked to aging-related cognitive decline.^30^ Interestingly, all these cytokines and chemokines are related to the IFN pathway: IL-18 is a main inducer of type II IFN (IFN-II),^31^ CXCL9 and CXCL10 are chemokines that are some of the most important IFN-responsive genes,^32^ and CCL11/eotaxin production is induced by IFN-I.^33^ This is a very important observation and consistent with prior studies showing that activation of IFN signaling drives inflammaging. For example, exaggerated IFN release has been associated with hematopoietic stem cell exhaustion,^34^ one of the main hallmarks of aging. De Cecco reported that the IFN-I response is a novel phenotype of late senescence.^35^ Rasa et al. demonstrated that upregulation of innate immune receptors and systemic IFN signaling underlies inflammaging across multiple tissues, which can be ameliorated by dietary restriction.^36^ Additionally, Arosio et al. showed that IFN-γ was significantly associated with the frailty index, a sensitive measure of physiological decline and biological age.^37^ These studies collectively support that chronic activation of IFN pathways contributes causally to biological aging and age-related functional decline. Our observation that the IFN pathway is also causally implicated in DNA methylation associated with aging is very important, as it further argues for an important role of this biological pathway as a potential therapeutic target for anti-aging interventions. Future studies should validate this concept in proof-of-principle clinical studies in humans, using IFN-modulating agents as a potential anti-aging approach.

Few studies have investigated the relationship between inflammation and EpiAge, with controversial results. Irvin et al. reported an association between classic markers of inflammation (CRP and IL-6) and Hannum EpiAge score,^38^ while Sanchez-Cabo and colleagues showed that the EAA calculated using the GrimAge score was associated with early atherosclerosis and increased concentrations of certain cytokines in the circulation (IL-6 and IL-10).^39^ Similarly, GrimAge age acceleration was associated with IL-1β, IL-6, and IL-8 concentrations in the lungs.^40^ In contrast, Cribb and colleagues argued that inflammation and EAA are broadly independent risk factors for biological aging, based on a lack of correlation between 22 biomarkers (classical inflammation markers and metabolites of the tryptophan pathway) and EpiAge scores.^41^ However, all these studies were limited by both a narrow characterization of inflammation and the correlational type of studies, which lack the capacity to infer conclusions on causality. We now performed a more comprehensive analysis, including MR studies that can provide causality information, of the association between inflammation and EAA based on the assessment of 92 biomarkers of inflammation in the circulation, out of which 64 biomarkers are measurable in a large majority of the individuals, and four of the main EpiAge scores. Our data strongly argue for an important role of inflammation, especially the IFN pathway, in EAA and, subsequently, age-related diseases. In contrast, and yet also very important for understanding the biology of aging, very little reverse causal effect has been observed between DNA methylation changes as a cause of inflammaging.

Finally, we also investigated the association between EAA and immune responsiveness to microbial ligands, as immunity declines with age, leading to increased susceptibility to infections and reduced response to vaccination.^42^ Our study highlights the impact of aging on cytokine production in response to pathogen stimulation, demonstrating potential age-related dysregulation and compensatory changes that may reflect underlying immune dysregulation. Consistent negative associations between IFN-γ production and age-related measures were observed in both cohorts, indicating that aging impairs T and/or natural killer (NK) cell response to microbial ligands. Similarly, a previous study also demonstrated reduced IFN-γ production in response to respiratory syncytial virus (RSV) in older individuals compared to younger individuals.^43^ IL-22 is primarily secreted by activated T cells and plays an important role in host defense against bacterial infection.^44^ The reduction in IL-22 production observed in the 500FG cohort further supports potential diminished T cell function with aging. An important point to be discussed is the apparent discrepancy between the IFN-mediated inflammation driving EAA (as described above) and the defective IFN-γ production in the elderly. Two aspects should be considered here: first, inappropriate continuous release of inflammatory mediators has often been associated with poor cytokine responsiveness upon microbial stimulation, a process previously described in individuals of older age.^45^ Second, it may well be possible that IFN-Is, rather than IFN-γ, drive the effects on EAA, and this remains to be investigated by future studies.

Positive associations between age-related measures and the production of IL-6, IL-10, and IL-1Ra align with the concept of inflammaging. Even though IL-10 and IL-1Ra are anti-inflammatory markers,^46^^,^^47^ they are most likely a compensatory response to counteract the heightened inflammatory milieu, thus reflecting the aging process. The strongest associations between age-related measures and PhenoAge or EAA_Pheno are consistent with a previous finding showing that accelerated DNA PhenoAge is linked to increased activation of pro-inflammatory pathways.^9^ Inflammaging contributes to the development of various age-related diseases.^48^ In our study, EAA_Grim showed the strongest association with frailty and the number of comorbidities, which is consistent with previous findings that GrimAge outperforms other epigenetic measures of age acceleration, such as Horvath, Hannum, and PhenoAge.^27^ Additionally, PhenoAge and EAA_Pheno were significantly correlated with frailty, aligning with earlier studies.^9^ Notably, EAA was significantly associated with COPD, supporting previous findings that accelerated epigenetic aging serves as a risk factor for COPD.^49^

Our study also has a few limitations. First, the inflammatory proteome assessment was performed using the inflammatory panel from Olink, which contains a relatively limited number of circulatory proteins (*n* = 92). Much larger biomarker arrays are now available, up to more than 5,000 proteins, and future studies should attempt to employ these approaches, although the costs of such measurements are still very high. Secondly, our cohorts all comprised individuals of European ancestry, and the results of the study cannot be automatically extended to other populations. More studies in non-European populations should be performed in the future. Finally, although our study was already performed in four independent cohorts, larger studies in elderly individuals with comorbidities should further strengthen and detail our findings in the future.

In summary, our study demonstrates that chronic inflammation, as assessed by circulating inflammatory proteins, especially those of the IFN pathway, causally contributes to biological age acceleration and common age-related diseases. EpiAge scores, especially those related to health span, such as GrimAge and PhenoAge, are very useful surrogates of biological aging associated with age-related diseases, and their use in epidemiological studies of aging can be very valuable. Future studies should investigate the molecular mechanisms through which CXCL9, CXCL10, CCL11, and IL-18 induce EAA and age-related diseases. These data argue for an important role of IFN pathway-driven inflammation in the pathophysiology of epigenetic alterations and biological aging in general. Therefore, future studies investigating IFN-targeted anti-aging prophylactic and therapeutic approaches are warranted.

## Resource availability

### Lead contact

Requests for further information should be directed to the lead contact, Mihai G. Netea (mihai.netea@radboudumc.nl).

### Materials availability

This study did not generate new reagents.

### Data and code availability

DNA methylation data have been deposited in the European Genome-phenome Archive (EGA), which is hosted by the EBI and the CRG, under accession numbers EGAS00001008031 (BCG-PRIME), EGAS00001008030 (iMED), 300BCG (EGAS00001007498), and EGAS00001008029 (500FG). Code for reproducing the plots, along with the necessary input files, is hosted on GitHub https://github.com/CiiM-Bioinformatics-group/BCG_prime_aging.git and Zenodo https://doi.org/10.5281/zenodo.18947285.^50^

## Acknowledgments

This study was partially supported by a Hevolution grant (#1332723) and an ERC Advanced Grant (#833247) (to M.G.N.). M.G.N. was supported by a Spinoza Grant of the Netherlands Organization for Scientific Research. C.-J.X. is supported by the Lower Saxony MWK Sprung Fund (19777006) and Deutsche Forschungsgemeinschaft (DFG) Fund (497673685). Y.L. is supported by an ERC Starting Grant (948207). We thank Dr. Javier Botey-Bataller for valuable discussions regarding the MR analysis.

## Author contributions

M.G.N. and C.-J.X. conceptualized and designed the study. Z.L. performed the data analysis supervised by C.-J.X., M.G.N., Y.L., and L.A.B.J. Y.Z. and N.v.U. helped with the validation of the MR findings, and M.K.G. implemented data pre-processing. M.B. helped with the raw data submission. A.Z., K.F., E.T., E.D., A.S., L.V., B.G. (BCG-PRIME), L.H., S.T., P.R., S.M., C.d.B., V.K., V.M., M.J. (300BCG and 500FG), F.P., and C.A.G. (iMED) helped with the recruitment of participants and biological data collection. Z.L., M.G.N., and C.-J.X. wrote the manuscript draft. All authors reviewed and approved the manuscript.

## Declaration of interests

M.G.N. is a scientific founder of Biotrip, Lemba, TTxD, and Salvina. L.A.B.J. is a scientific founder of Lemba, TTxD, and Salvina.

## STAR★Methods

### Key resources table

**Table:** 

REAGENT or RESOURCE	SOURCE	IDENTIFIER
**Bacterial and virus strains**		

Heat-inactivated SARS-CoV-2 Wuhan-Hu-1 variant	Provided by Heiner Schaal	N/A
Heat-inactivated SARS-CoV-2 Omicron BA.1 variant	Provided by Heiner Schaal	N/A
Heat-inactivated SARS-CoV-2 Delta variant	Provided by Heiner Schaal	N/A
Heat-inactivated influenza A (H1N1) virus	provided by Ortwin Adams	N/A
Heat-killed Candida albicans	ATCC	UC820
R848	InvivoGen	N/A
Purified lipopolysaccharide (LPS)	Sigma-Aldrich	Escherichia coli O55:B5

**Biological samples**		

Human whole blood	This paper	N/A
Human peripheral blood mononuclear cell	This paper	N/A

**Critical commercial assays**		

Human IL-1 beta ELISA	R&D systems	DY201
Human IL-6 ELISA	R&D systems	DY206
Human TNF ELISA	R&D systems	DY210
Human IL-10 ELISA	R&D systems	DY217B
Human IL-1RA ELISA	R&D systems	DY280
Human IFN-gamma ELISA	R&D systems	DY285B
Human IFN-alpha ELISA	Mabtech	3425-1H-6

**Deposited data**		

DNA methylation data(BCG-PRIME)	This paper	EGAS00001008031
DNA methylation data(iMED)	This paper	EGAS00001008030
DNA methylation data(300BCG)	This paper	EGAS00001007498
DNA methylation data(500FGd)	This paper	EGAS00001008029
GWAS of epigenetic age acceleration	McCartney et al.^18^	https://datashare.is.ed.ac.uk/handle/10283/3645
pQTL	Sun et al.^17^	http://ukb-ppp.gwas.eu

**Software and algorithms**		

Code for statistical analysis and generating plot	This paper^50^	https://github.com/CiiM-Bioinformatics-group/BCG_prime_aging.git and https://doi.org/10.5281/zenodo.1894728555
R version 4.2.0	R Core Team	https://www.r-project.org/
R package minfi version 4.2.0	Aryee et al.^51^	https://github.com/hansenlab/minfi
DNA methylation age calculation	Horvath et al.^7^	https://dnamage.clockfoundation.org
R package TwoSampleMR version 0.6.20	Hemani et al.^16^	https://mrcieu.github.io/TwoSampleMR/

### Experimental model and study participant details

For this study, we included four independent cohorts:

(1) BCG-PRIME cohort: Participants were recruited from community-dwelling adults aged 60 years or older with at least one underlying comorbidity. This cohort was originally established for a randomized controlled trial, in which participants were randomized 1:1 to receive either BCG or placebo vaccination, as reported in Koekenbier et al. 2023.^52^ For the present study, we exclusively used baseline samples collected at Radboud University Medical Center, Nijmegen, the Netherlands, prior to the BCG or COVID-19 vaccinations that were employed in the study. A total of 384 participants (males = 243, females = 141), with a mean age of 69 years (±5 SD) and one or more underlying comorbidities, were included. All participants had available data on whole genome-wide DNA methylation profiles, circulating inflammatory proteins, and cytokine production capacity in response to various pathogen stimulations. All participants provided written informed consent before undergoing any study procedures. The trial was approved by the Utrecht Institutional Review Board (protocol NL74730.041.20), registered in the European Clinical Trials Database (2020-003470-47), and conducted in compliance with the ethical principles of the Declaration of Helsinki and the Guidelines for Good Clinical Practice.
(2) iMed cohort: This cohort was established as part of a population-based study involving individuals aged ≥65 years from Hannover, Germany, geared toward studying immune responses to influenza vaccination in this age group. Detailed descriptions of this cohort have been provided in prior studies.^53^^,^^54^^,^^55^^,^^56^ Blood samples collected before vaccination (Baseline) were included in the present study. A total of 165 participants (males = 90, females = 75) with a mean age of 72 years (±4 SD) were included in this study. All participants had available data on whole genome-wide DNA methylation profiles and circulating inflammatory proteins. This cohort was approved by the ethics committee (ref no. 6775, dated 15 September 2015) of Hannover Medical School and the study was funded by iMed, the Helmholtz Association’s Cross Program Initiative on Personalized Medicine. All participants provided written informed consent prior to undergoing any study procedures.
(3) 300BCG cohort: This cohort was established with healthy adult volunteers of Western European ancestry, recruited between April 2017 and June 2018 at Radboud University Medical Center, Nijmegen, The Netherlands. Detailed descriptions of this cohort have been provided in prior studies.^57^ For the present study, 283 participants (males = 126, females = 157), with a mean age of 25 years (±10 SD), were included. These participants had available data on whole genome-wide DNA methylation profiles and circulating inflammatory proteins. The 300BCG study was approved by the Arnhem-Nijmegen Medical Ethical Committee with number NL58553.091.16. All studies were performed in accordance with the declaration of Helsinki. All participants provided written informed consent prior to undergoing any study procedures.
(4) 500FG cohort: This cohort was established with healthy adult volunteers of Western European ancestry, recruited between August 2013 and December 2014 at Radboud University Medical Center, Nijmegen, The Netherlands. For the present study, a total of 212 participants (males = 104, females = 108) were included, with a mean age of 25 years (±11 SD). All participants had available data on whole genome-wide DNA methylation profiles, circulating inflammatory proteins, and cytokine production in response to various pathogen stimulations. The study was approved by the Ethical Committee of Radboud University Medical Center Nijmegen (NL42561.091.12, 2012/550). The inclusion of volunteers and experiments were conducted according to the principles expressed in the Declaration of Helsinki. All volunteers gave written informed consent before any material was taken.

### Method details

#### DNA methylation profiling

DNA was purified from whole blood using QIAamp DNA blood kits (Qiagen Benelux BV, Venlo, the Netherlands). DNA concentration was quantified with a NanoDrop spectrophotometer at 260 nm. High-quality DNA was utilized for genome-wide DNA methylation profiling, conducted by either Infinium© MethylationEPIC array (BCG cohort) (∼850,000 CpG sites) or MethylationEPIC v2 array (500FG, iMed, and BCG-PRIME cohorts) (∼937,055 CpG sites).

#### Circulating proteome measurements

Circulating plasma inflammatory markers were measured using the commercially available Olink Proteomics AB Inflammation Panel (92 inflammatory proteins), using multiplex proximity extension assay.^58^ In this assay, proteins are recognized by pairs of antibodies coupled to cDNA strands, which bind when they are in close proximity and extend by a polymerase reaction. A pooled plasma sample and an interplate control were included on each plate in triplicate to correct for batch differences. Detected proteins were normalized according to interplate controls to minimize interassay variation and measured on a log2 scale as normalized protein expression values.

#### Cytokine production capacity measurement

For the 500FG cohort, details of stimulation experiments and cytokine measurements were described previously.^59^ Briefly, whole blood was diluted with pyrogen-free saline, followed by PBMC isolation using density gradient centrifugation with Ficoll-Paque (Pharmacia Biotech, Uppsala). The PBMC fraction was collected, washed twice with saline, and resuspended in RPMI 1640 medium supplemented with gentamicin (10 mg/mL), L-glutamine (10 mM), and pyruvate (10 mM). 5 × 10^5^ PBMCs in a final volume of 200 μL medium per well were incubated at 37°C in round-bottom 96-well plates with different stimuli. Supernatants were collected after 24 h for IL-1β, TNF, IL-6 measurement or after 7 days for IFN-γ, IL-22, and IL-17 measurement. Cytokine concentrations were determined using specific commercial ELISA kits (PeliKine Compact, Amsterdam, or R&D Systems) according to the manufacturer’s instructions.

For the BCG-PRIME cohort, PBMCs were isolated from EDTA anticoagulated blood using gradient centrifugation over Ficoll-Paque Plus (GE Healthcare) in SepMate tubes (STEMCELL Technologies), following the manufacturer’s instructions. The isolated cells were cryopreserved in Recovery cell culture freezing medium (Thermo Fisher Scientific) at −150°C until analysis. PBMCs were thawed, and washed twice with Dutch-modified RPMI medium (Thermo Fisher Scientific) supplemented with gentamicin (50 μg/mL), GlutaMAX (2 mM), pyruvate (1 mM), and 10% fetal bovine serum (FCS; Fischer Scientific) and resuspended in Dutch-modified RPMI supplemented with gentamicin (50 μg/mL), GlutaMAX (2 mM), pyruvate (1 mM), and 10% human pooled serum. 5 × 10^5^ PBMCs were cultured in a final volume of 200 μL per well in round-bottom 96-well plates and stimulated with heat-inactivated SARS-CoV-2 Wuhan-Hu-1 variant (8.9 × 10^4^ TCID_50_/mL; NRW-42 isolate), heat-inactivated SARS-CoV-2 Omicron BA.1 variant (7.7 × 10^3^ TCID_50_/mL), heat-inactivated SARS-CoV-2 Delta variant (8.9 × 10^2^ TCID_50_/mL) (all kindly provided by Heiner Schaal, University Hospital Düsseldorf, Germany), heat-inactivated influenza A (H1N1) virus (3.37 × 10^7^ K/mL, kindly provided by Ortwin Adams, University Hospital Düsseldorf, Germany), heat-inactivated Staphylococcus aureus (1 × 10^6^ CFU/mL; clinical isolate), heat-killed Candida albicans (1 × 10^6^ CFU/mL; UC820; ATCC), R848 (1 μg/mL; InvivoGen), or purified lipopolysaccharide (LPS) derived from Escherichia coli O55:B5 (10 ng/mL; Sigma-Aldrich), and incubated for 48 h at 37°C with 5% CO_2_. Supernatants were collected after 48 h and stored at −20°C for subsequent analysis. Cytokine concentrations in PBMC culture supernatants were measured using ELISA, following the manufacturer’s instructions. IL-1β, TNF, IL-6, IL-1Ra, IL-10, and IFN-γ concentrations were determined using DuoSet ELISA kits (R&D Systems). The ELISA Flex: Human IFN-α (HRP) (Mabtech) was used for IFN-α measurement.

### Quantification and statistical analysis

#### DNA methylation quantification and quality control

The DNA methylation values were obtained from the raw IDAT files using the minfi R package (v.4.2.0).^51^ Poor quality samples and those with sex-mismatched were excluded. Stratified quantile normalization^60^ was applied after filtering out bad quality probes with a detection *p* value >0.01, cross-reactive probes, polymorphic probes,^61^ and probes located on the sex chromosomes. Problematic probes due to mapping inaccuracies and flagged probes in the EPIC v2 array provided by Illumina were also removed in the 500FG cohort, iMed, and BCG-PRIME cohorts.

#### DNA methylation age calculation

DNA methylation beta values were obtained using the getBeta function from the minfi R package. DNA methylation age was calculated through the online calculator portal, following the instructions provided (https://dnamage.clockfoundation.org).^7^ DNA methylation age (epigenetic age, EpiAge) acceleration (EAA) was calculated as the residual from a regression analysis of DNA methylation age on chronological age, using an R model: residuals (lm(EpiAge ∼ Age)).

#### Association analyses

We investigated the associations between age-related measures (Age, EpiAge, and EAA) and inflammatory proteins, cytokines, and clinical phenotypes using the following models: (1) The associations between age-related phenotypes and proteins or cytokines were assessed using linear regression models: protein/cytokine ∼ age-related phenotypes + sex. Cytokine and protein values were rank-normalized. Sex was included as a covariate. (2) The associations between age-related phenotypes and binary comorbidity outcomes (yes/no) were evaluated using generalized linear models as: comorbidity (yes/no) ∼ age-related phenotypes + sex and comorbidity (yes/no) ∼ age phenotypes + sex + smoking. (3) For continuous clinical outcomes such as the number of comorbidities or frailty scores (Y), associations were analyzed using linear regression models as follows: Y ∼ age-related phenotypes + sex.

#### Two-sample Mendelian randomization

We obtained summary statistics from two publicly available genome-wide association studies (GWASs): one on epigenetic age acceleration (EAA)^18^ and the other on protein levels.^17^ Both GWASs were derived from European cohorts, and there is no overlap between these two cohorts. For significant protein-EAA associations identified in this study, the causal inference was further explored using bidirectional two-sample MR analysis with instruments of proteins and EAA. Instruments to proxy for protein abundance were variants associated in cis (±500,000 bp of the transcription start sites) and in trans, separately, at genome-wide significance (*p* < 5e−08) extracted from the protein genome-wide association study (pQTL) summary statistics.^17^ Instruments to proxy for EAA incidence were variants at *p* < 5e−08 extracted from the EAA GWAS summary statistics.^18^ Linkage disequilibrium clumping (r2 < 0.001) was conducted using the European 1000 Genomes Project phase 3 as the reference panel to ensure the independence of the instrumental variables. We removed instruments with an F-statistic of less than 10 to reduce weak instrument bias. The Wald ratio was used to estimate MR effects if only a single instrument was available, and the inverse variance weighted (IVW) method was used if two or more instruments were available. We defined two kinds of relationship between proteins and EAA, as detailed in the results. First, a protein was causally associated with EAA, which is evidenced by a significant association (*p* < 0.05) identified in the protein-to-EAA direction as well as an insignificant association revealed in the EAA-to-protein direction. Second, the altered plasma level of a protein was a consequence of EAA, as evidenced by a significant association in the EAA-to-protein direction while insignificant in the protein-to-EAA direction. MR analyses were performed using the mr function from the TwoSampleMR^16^ package(v0.6.20) in R(v.4.2.0). For Mendelian randomization with significant IVW (*p* < 0.05), sensitivity analyses were performed, including testing for horizontal pleiotropy, heterogeneity, and leave-one-out. For the MR results with IVW significance, other methods were also used to test their significance, including Weighted median, Weighted mode, and MR-PRESSO. The number of SNP instruments per protein (reported only for proteins with significant MR results) and the average F-statistics are shown in Table S6.

#### Enrichment analysis

To identify potential upstream regulators, enrichment analysis was performed using the enrichR package against the following transcription factor–target databases: ChEA 2022, ENCODE TF ChIP-seq 2015, and TRRUST Transcription Factors 2019. Significant transcription factors were defined based on *p*adj <0.05. For visualization, −log10(*p*) values were calculated, and the top enriched transcription factors were visualized based on statistical significance.

To evaluate whether interferon-stimulated genes were overrepresented among proteins significantly associated with epigenetic age acceleration, proteins with an adjusted *p* value <0.05 were first identified. Olink protein identifiers were mapped to official gene symbols using standardized gene annotation. The ISG gene set was constructed based on the canonical interferon-responsive gene set (*CD274, TNFSF10, CCL2, CCL8, IL12B, IFI44, IFI44L, IFIT1, IFIT2, IFIT3, ISG15, MX1, OAS1, OAS2, OAS3, RSAD2, CXCL9, CXCL10, CXCL11, IFNG, STAT1, IL18R1, IL10RB, CD40, CCL4*). Enrichment was assessed using a two-sided Fisher’s exact test based on a 2 × 2 contingency table comparing ISG status (ISG vs. non-ISG) and significance status (significant vs. non-significant proteins). To further validate enrichment robustness, a permutation-based approach was performed. Specifically, 10,000 random protein sets equal in size to the observed significant protein list were sampled from the full tested protein background. For each iteration, the number of ISGs was recorded. The empirical *p* value was calculated as the proportion of permutations yielding an equal or greater number of ISGs than observed (right-tailed test).

## Supplemental information

Document S1. Figures S1−S4 and Tables S5 and S6

Table S1. The correlation results between inflammatory proteins and aging scores in the BCG-PRIME cohort, related to Figure 3

Table S2. The correlation results between inflammatory proteins and aging scores in the iMED cohort, related to Figure 3

Table S3. The correlation results between inflammatory proteins and aging scores in the 300BCG cohort, related to Figure 3

Table S4. The correlation results between inflammatory proteins and aging scores in the 500FG cohort, related to Figure 3

Document S2. Article plus supplemental information
