# Supplementary material for: Interferon-related inflammaging links epigenetic age acceleration to multimorbidity
Source: Cell Genom. 2026 Apr 17;6(6):101218. doi: 10.1016/j.xgen.2026.101218 (PMC13261661; doi:10.1016/j.xgen.2026.101218)
Supplement: Document S1. Figures S1−S4 and Tables S5 and S6 [file mmc1.pdf]

Cell Genomics, Volume *6*

Supplemental information

Interferon-related inflammaging links epigenetic age acceleration to multimorbidity

Zhaoli Liu, Athanasios Ziogas, Yihan Zhang, Manoj Kumar Gupta, Konstantin Föhse, Esther Taks, Elisabeth Dulfer, Andrei Sarlea, Lorenzo Ventriglia, Büsra Geckin, Mohamad Ballan, Nienke van Unen, Leonie Helder, Stephanie Trittel, Peggy Riese, Simone Moorlag, Charlotte de Bree, Valerie Koeken, Vera Mourits, Martin Jaeger, Frank Pessler, Carlos A. Guzmán, Leo A.B. Joosten, Yang Li, Cheng-Jian Xu, and Mihai G. Netea

Figure S1

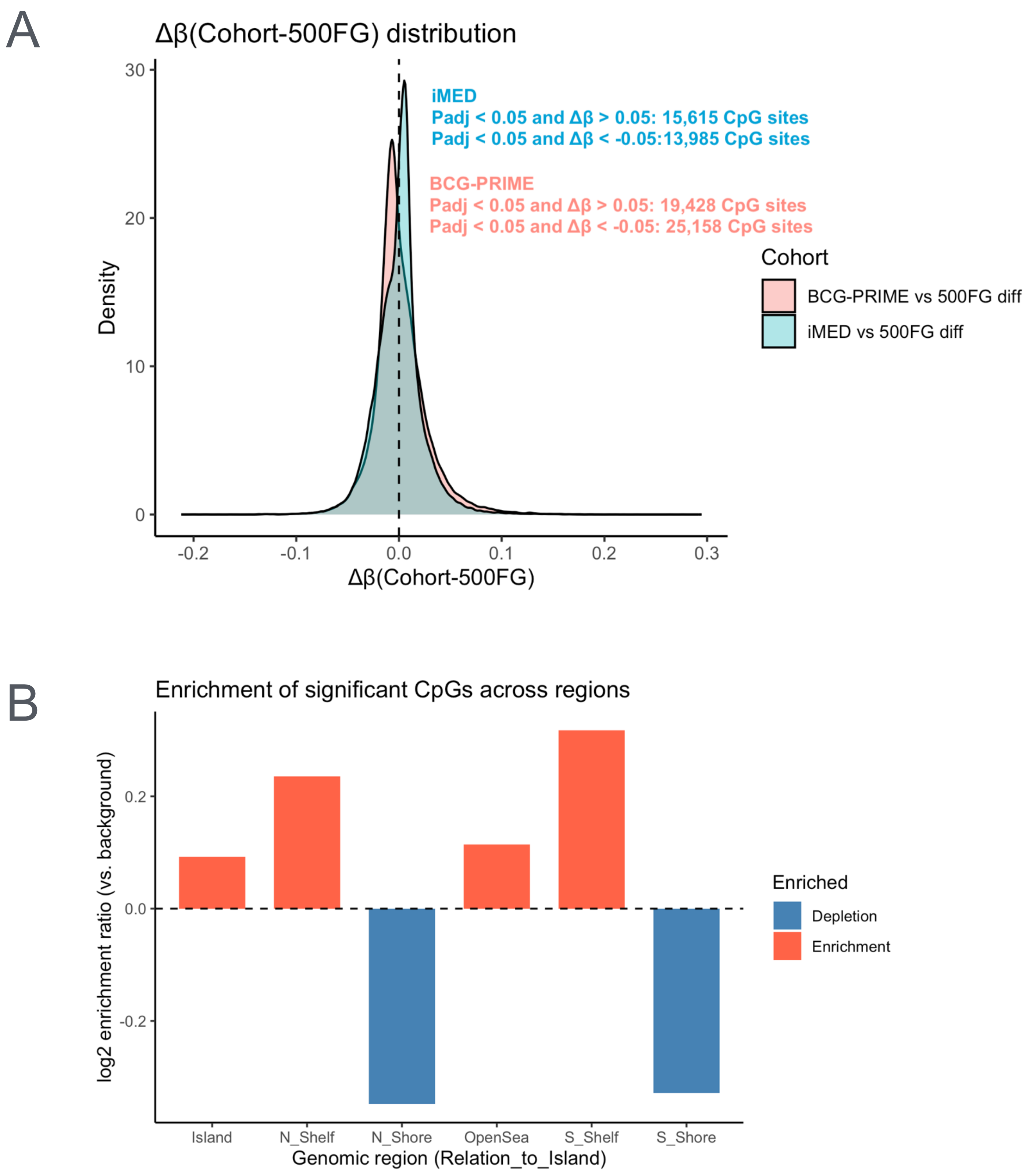

Figure S1: Comparative DNA methylation profiles and genomic region enrichment across cohorts, related to Figure 1. A. Distribution of methylation differences (Δβ) across cohorts relative to 500FG. Density plots show the distribution of average methylation differences (Δβ) for CpG sites included in the epigenetic aging clock models, comparing each cohort (BCG-PRIME and iMED) to the 500FG reference cohort. The Δβ values were computed as the difference in mean β-values between each cohort and 500FG. Distributions are centered around zero, suggesting no consistent global hyper- or hypomethylation across cohorts. B. Enrichment of significant CpG sites across genomic regions. Bar plot showing $\log_2$ enrichment ratios of significantly differentially methylated CpG sites (FDR < 0.05, |Δβ| > 0.05) across genomic regions defined by CpG island context (Island, Shore, Shelf, OpenSea). Enrichment ratios were calculated relative to the background distribution of all tested CpGs. Bars above zero (in red) indicate enrichment, while bars below zero (in blue) indicate depletion.

Figure S2

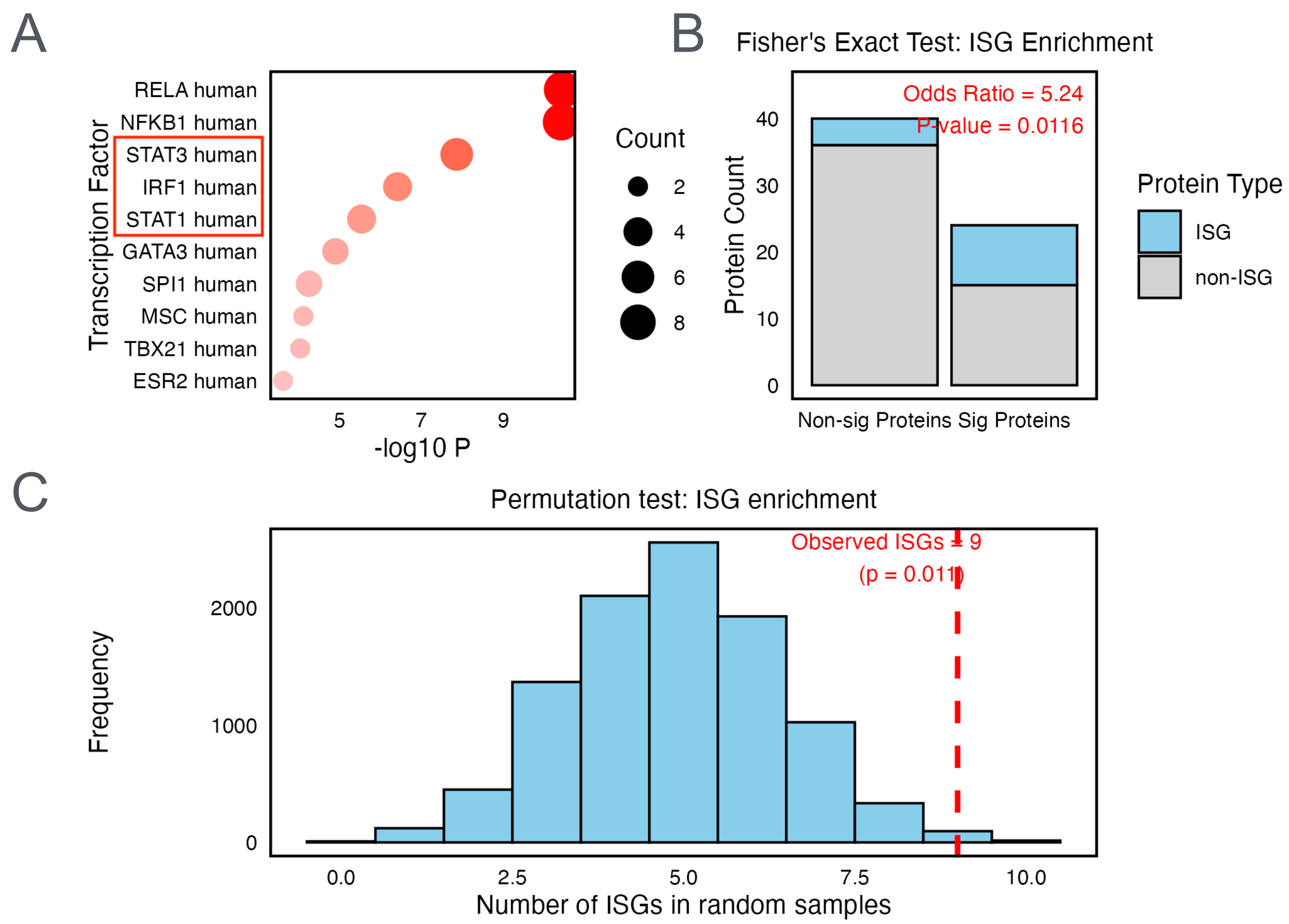

Figure S2: Interferon-related regulatory and gene set enrichment analyses of EAA-associated proteins, related to STAR Methods. A. Transcription factor enrichment analysis of proteins associated with epigenetic age acceleration. IRF and STAT family members, including IRF1, STAT1, and STAT3, were significantly enriched as upstream regulators, indicating activation of interferon-related transcriptional programs. B. Enrichment of interferon-stimulated genes (ISG) among EAA-associated proteins assessed by Fisher's exact test. The proportion of ISGs was higher in the significant protein set compared to the background proteome. C. Permutation-based validation of ISG enrichment. The observed number of ISGs among significant proteins (red dashed line) exceeded the distribution obtained from 10,000 random samplings of proteins of equal size, supporting a non-random enrichment of interferon-responsive genes.

Figure S3

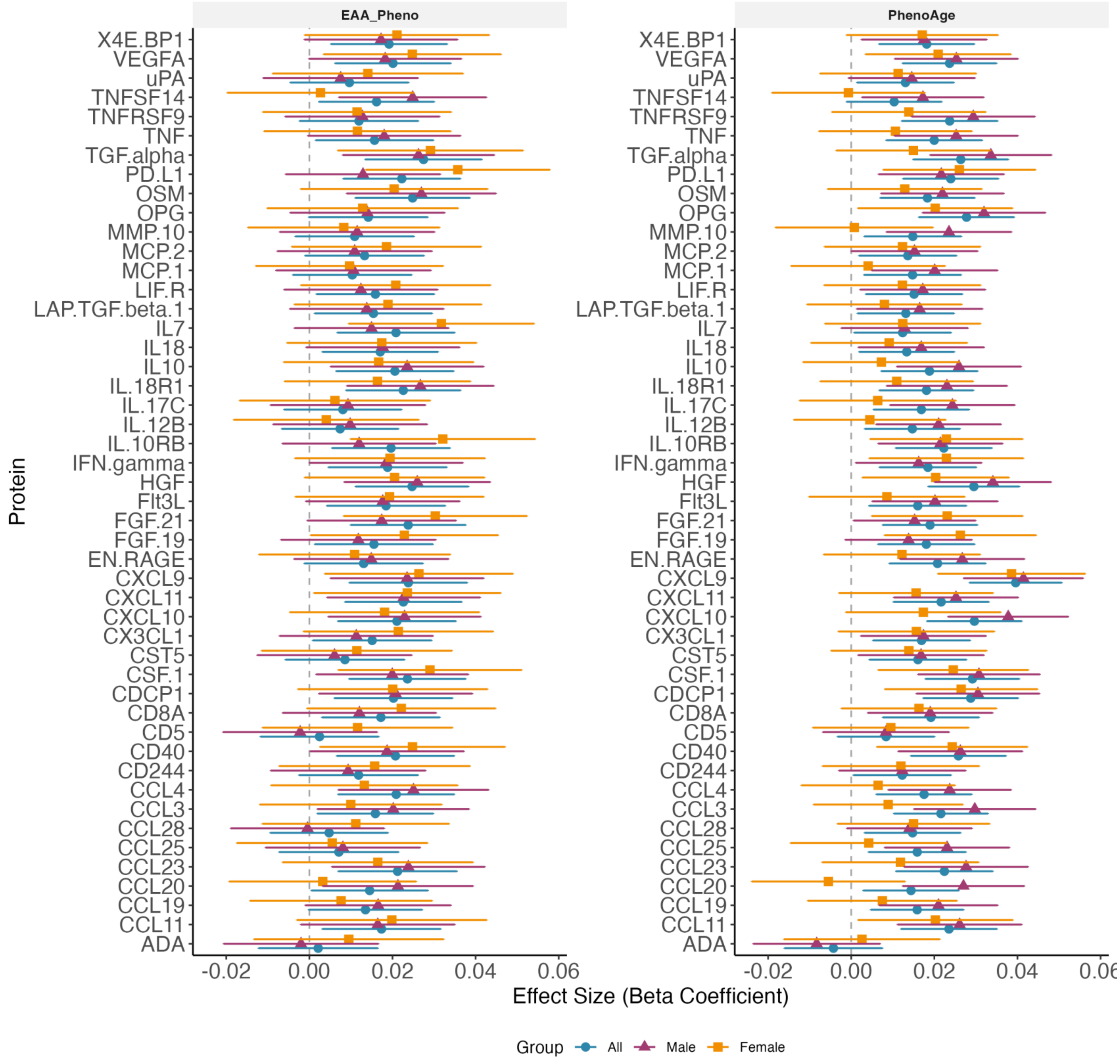

Figure S3: Sex-stratified analysis of the associations between inflammatory proteins and Phenoage or EAA_Pheno, related to Figure 5. Forest plots showing the effect sizes (beta coefficients) and 95% confidence intervals from the linear regression models for the overall cohort, males, and females.

Figure S4

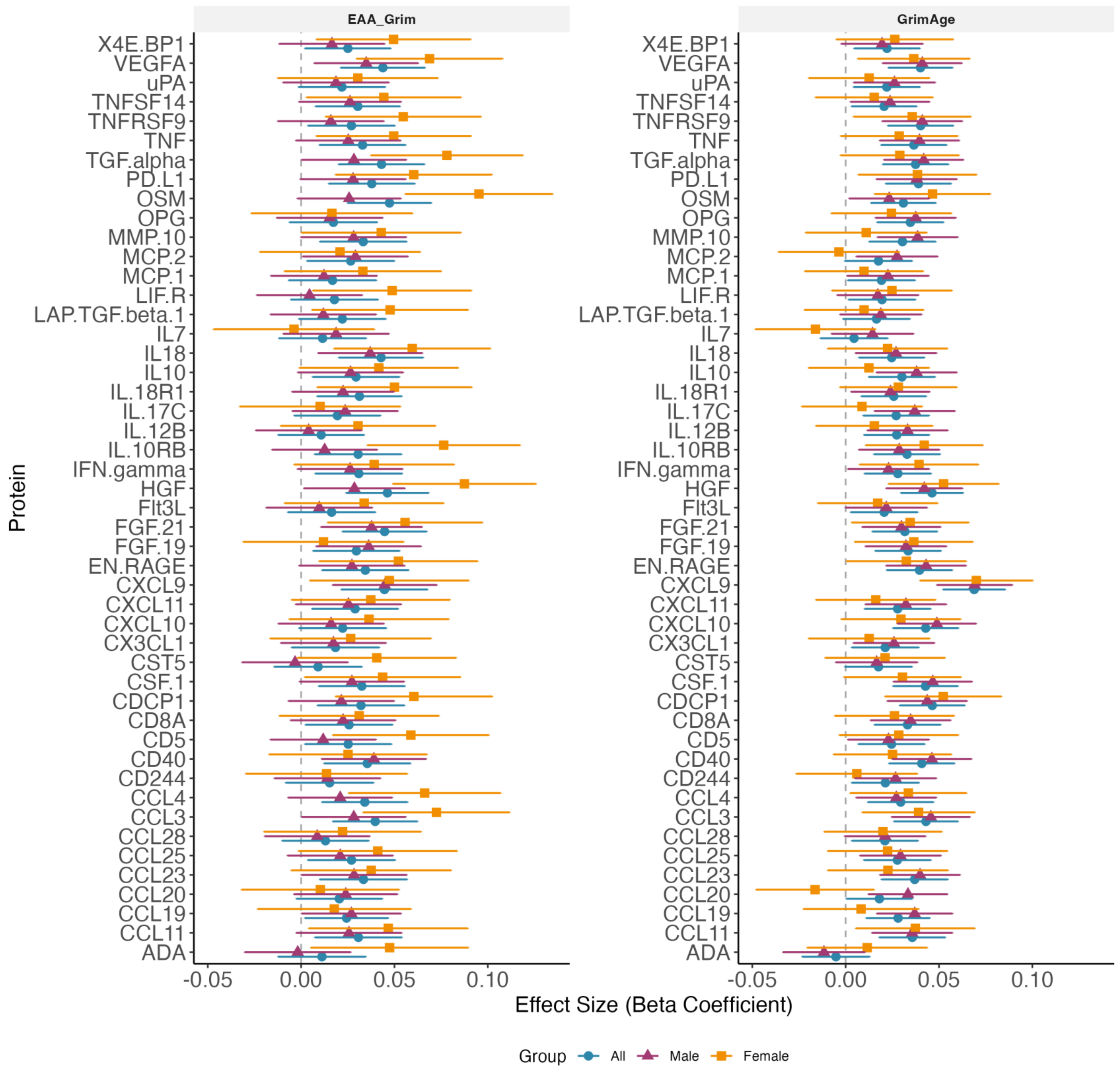

Figure S4: Sex-stratified analysis of the associations between inflammatory proteins and Grimage or EAA_Grim, related to Figure 5. Forest plots showing the effect sizes (beta coefficients) and 95% confidence intervals from the linear regression models for the overall cohort, males, and females.

Table S5

outcome	protein	b	se	pval	padj
EAA_Pheno	CXCL9	0.561996	0.154261	**0.000269**	**0.006733**
EAA_Pheno	CXCL10	0.476638	0.189204	0.011763	0.14704
EAA_Pheno	CCL4	0.203404	0.09198	0.027009	0.186535
EAA_Pheno	CCL11	0.254719	0.120098	0.033927	0.186535
EAA_Pheno	IL18	0.300184	0.1512	0.047107	0.196279
EAA_Grim	CXCL10	0.359408	0.162409	0.026899	0.224157
EAA_Grim	CXCL9	0.486251	0.134409	**0.000297**	**0.007431**
EAA_Grim	CCL11	0.207958	0.103067	0.043623	0.272646

Table S5: Protein–EAA pairs showing nominal evidence of potential causal relationships in the Mendelian randomization analysis, related to Figure 4.

Table S6

protein	EAA_Grim trans MR	snp before clumping (P < 5e-08)	snp_after_clumping	snp after filtering by F > 10	mean_F_after_filter	median_F	min_F	max_F
CCL11	EAA_Grim trans MR	2705	22	17	117.6422844	53.1229086	29.7181575	762.790858
CCL4	EAA_Grim trans MR	5256	30	27	165.1411428	52.0529088	30.4341868	1259.04877
CXCL10	EAA_Grim trans MR	4008	17	13	66.181095	42.5636084	32.2180291	204.778488
CXCL9	EAA_Grim trans MR	5785	18	17	84.24964407	39.3752801	33.3787074	345.079851
IL18	EAA_Grim trans MR	4336	27	18	113.9927652	52.9427491	31.5073729	852.05233
CCL11	EAA_Pheno trans MR	2705	22	17	117.6422844	53.1229086	29.7181575	762.790858
CCL4	EAA_Pheno trans MR	5256	30	27	165.1411428	52.0529088	30.4341868	1259.04877
CXCL10	EAA_Pheno trans MR	4008	17	13	66.181095	42.5636084	32.2180291	204.778488
CXCL9	EAA_Pheno trans MR	5785	18	17	84.24964407	39.3752801	33.3787074	345.079851
IL18	EAA_Pheno trans MR	4336	27	18	113.9927652	52.9427491	31.5073729	852.05233
CXCL10	EAA_Grim cis MR	699	4	3	61.18414097	49.6960163	38.6766492	95.1797574
CXCL9	EAA_Grim cis MR	850	2	2	202.8333646	202.833365	60.5868777	345.079851
TNF	EAA_Grim cis MR	2619	4	1	136.2098773	136.209877	136.209877	136.209877
CXCL9	EAA_Pheno cis MR	850	2	2	202.8333646	202.833365	60.5868777	345.079851

Table S6: The number of SNP instruments per protein (reported only for proteins with significant MR results) and the average F-statistics, related to Figure 4.
